# Metabolic profiling analysis of cytochrome *b*_*5*_ production in *E. coli * N4830-1 using GC-MS

**DOI:** 10.1007/s11306-026-02522-5

**Published:** 2026-09-22

**Authors:** Thanyaporn Tengsuttiwat, Adam Burke, Naheed Nazly Kaderbhai, Joe Gallagher, Howbeer Muhamadali, Royston Goodacre

**Affiliations:** 1 Department of Biochemistry, Cell and Systems Biology, Centre for Metabolomics Research, Institute of Systems, Molecular and Integrative Biology, University of Liverpool, Liverpool, L69 7ZB UK; 2 National Center for Genetic Engineering and Biotechnology (BIOTEC), National Science and Technology Development Agency (NSTDA), Khlong Nueng, Khlong Luang, Pathumthani, 12120 Thailand; 3 Institute of Biological, Environmental and Rural Sciences, Aberystwyth University, Gogerddan Campus, Penglais, Aberystwyth, Ceredigion SY23 3FL UK

**Keywords:** GC-MS, Metabolic profile, Recombinant protein production, Cytochrome *b*_5_, Chemometrics

## Abstract

**Introduction:**

Recombinant protein production is pivotal across diverse industries, necessitating efforts to enhance both quantity and quality.

**Objectives:**

We employed gas chromatography coupled with mass spectrometry (GC-MS) to investigate the metabolic effects of producing mammalian cytochrome *b*_5_ in the bacterial host *E. coli* N4830-1. The model system studied involved the cyt *b*_5_ gene being introduced on plasmids into the host with varying copy numbers (0–6) under a *λP*_L_ heat-sensitive promoter.

**Methods:**

Metabolic profiling involved GC-MS analysis with quality assessments using pooled QCs and multivariate chemometric analysis. This approach revealed specific metabolic features (identified to Level 2 of the Metabolomics Standards Initiative ) correlating significantly with different cultivation conditions and variable copy numbers of the cyt *b*_5_ gene among the examined bacterial strains.

**Results:**

Our study shows that CYT *b*_5_ production imposed a substantial energetic burden, reflected by depletion of metabolites associated with the tricarboxylic acid cycle, glycolysis/gluconeogenesis, pentose phosphate pathway, and glyoxylate metabolism, indicating increased ATP demand. Concurrent reductions in unsaturated fatty acids and changes in lipid-related metabolites suggest membrane remodeling in response to temperature induction (needed to initiate production of CYT *b*_5_), as well as metabolic stress. Alterations in glyoxylate shunt and pentose phosphate pathway intermediates further indicate metabolic reprogramming to maintain carbon homeostasis, while significant changes in nucleotide- and amino acid-related metabolites suggest impacts on biosynthetic capacity associated with recombinant expression and gene copy number variation.

**Conclusion:**

Overall, these findings demonstrate that CYT *b*_5_ production induces coordinated metabolic adjustments affecting energy-related metabolic pathways, bacterial cell wall and membrane biosynthesis, nucleotide metabolisms and bacterial stress responses, providing valuable insights that may be useful for the optimisation of recombinant protein production processes.

**Supplementary Information:**

The online version contains supplementary material available at https://doi.org/10.1007/s11306-026-02522-5.

## Introduction

Recombinant protein production has played a crucial role for decades in a broad range of industries; i.e., pharmaceuticals, food, and cosmetics (Kirk et al., 2002; Singh et al., 2016). The recombinant proteins market is expanding rapidly, projected to grow from about USD 3.25 billion in 2024 to nearly USD 8.66 billion by 2034, reflecting strong global demand. This growth underscores the critical role of recombinant protein technologies in modern biopharmaceuticals, including vaccines, therapeutic enzymes, and monoclonal antibodies (BioSpace, 2024). Although there are certain known limitations due to the lack of the machinery required for post-translational modification of eukaryotic proteins (Prabhu et al., 2021), *E. coli* is still a popular host. It offers numerous advantages, including ease of handling, genetic tractability, minimal growth requirements, rapid growth, and extensive development over many years making it a versatile host suitable for a wide range of applications (Leis et al., 2013). However, the development of improved bacterial hosts for the expression of recombinant proteins more efficiently is still an area of interest with strategies based on the optimisation of culturing conditions and genetic engineering methods (Tripathi & Shrivastava, 2019).

The temperature-inducible λ *c*I857/*λP*_L_ system provides a tightly regulated, chemically independent and synchronous induction mechanism for recombinant protein expression in *E. coli*, ensuring minimal basal expression at 30 °C prior to induction. This system has been widely used for controlled expression of heterologous proteins and is historically linked to the thermo-inducible CYT *b*_5_-based reporter platform developed by Kaderbhai *et al*. (1992), from which the expression strain and induction framework used in the present study were originally derived.

Over the last two decades, metabolomics has emerged as a valuable tool for refining recombinant expression systems. By investigating the metabolome, which encompasses the fundamental constituents of diverse biomolecules involved in metabolism, including amino acids that are the building blocks of proteins, metabolomics offers insights into the biochemical alterations within the system that may not be fully elucidated through genomic approaches (Fiehn, 2002). Metabolomics can be described as the study of low molecular weight compounds which are usually primary metabolites involved in necessary bioprocesses that are essential for the maintenance and growth of cells (Dunn & Ellis, 2005; Hollywood et al., 2006; Mashego et al., 2007). The synthesis of secondary metabolites necessitates energy, such as ATP, as well as precursors, cofactors, and other pertinent building blocks, which are derived from basic primary metabolites (Bailey, 1991). Thus, investigating the correlation between primary and secondary metabolites and recombinant protein production levels, along with low molecular weight compounds, is imperative, as the availability of these metabolites may directly impact the biosynthetic rate of the desired recombinant protein (Stephanopoulos & Vallino, 1991). Additionally, given that the presence and maintenance of foreign DNA can influence both the quality and quantity of the recombinant protein, comprehending the metabolic burdens associated with harbouring such DNA material through metabolomics is beneficial for enhancing the recombinant protein production process (Muhamadali et al. 2015a, 2016).

To monitor recombinant protein expression, Kaderbhai developed a reporter system in which positive expression within a microbial host is detected spectrophotometrically through a visible transformation to a bright pink colour (Kaderbhai et al., 1992). Building on this foundation, our previous study investigated cytochrome *b*_*5*_-producing *E. coli* N4830-1 using metabolomic fingerprinting via Fourier transform infrared (FT-IR) spectroscopy (Tengsuttiwat et al., 2022). While FT-IR spectroscopy provides rapid metabolic fingerprints, more detailed metabolite profiling is required to elucidate pathway-level alterations associated with recombinant protein production. Mass spectrometry (MS) – especially when coupled with chromatographic separation methods is frequently used in metabolomics due to its high sensitivity, selectivity, accuracy and resolution (Kaderbhai et al., 2003; Dunn & Ellis, 2005; Dunn et al., 2011a, 2011b; Dunn et al., 2013). Therefore, the objective of this study was to characterise the metabolic alterations associated with recombinant CYT *b*_5_ production in *E. coli* using GC-MS-based metabolomics coupled with chemometric analysis. By identifying metabolites and metabolic pathways perturbed during recombinant protein expression, this study aims to provide mechanistic insights into the metabolic burden imposed by recombinant protein production and to identify potential targets for improving expression efficiency.

## Methods

### Bacterial strains and culture conditions

Seven strains of *Escherichia coli* N4830-1 (F^−^*suo thi-1 thr-1 leuB6 lacY1 fhuA21 supE44 rfbD1 mcrA1 his ilv galK8* Δ(*hemF-exp*) Δ(*bio uvrB*) [λ Δ*Bam* N^+^*c*I857 Δ(CroattR)]) harbouring the pEX-CYT plasmid were kindly provided by Dr Naheed Kaderbhai. The strains carry different copy numbers of cyt *b*_5_ gene, ranging from 0 to 6, which are labelled as N0 to N6. All strains were streaked onto LB agar (Formedium, UK) prior to growing overnight at 37 °C with 75 µg/mL ampicillin supplemented LB agar for selection of the desired colonies. The cells were cultured for the CYT *b*_5_ production as described by (Kaderbhai et al., 1992; Tengsuttiwat et al., 2022). Briefly, 1 mL overnight culture was inoculated into a 250 mL Erlenmeyer flask containing 25 mL of LB medium with ampicillin (75 µg/mL). The cells were first incubated shaking at 150 rpm and 30 °C for 2.5 h to reach the mid-log phase, the incubation temperature was then immediately increased to 38.5 °C to activate the promoter, *λP*_L_. The culture was incubated for 9 h to allow for the protein production at 38.5 °C, while a control set (N0-N6) was incubated for the same duration at 30 °C.

### Sample preparation

After 9 h of cultivation, each of the bacterial samples were collected as 1 mL aliquots to determine their biomass by measuring the optical density at 600 nm (OD_600nm_) which was subsequently used for biomass normalisation. The bacterial samples were also collected for metabolic profiling by immediately halting the metabolic process using solvent-based quenching, following which bacterial metabolites were subsequently extracted from the samples using cold methanol. Derivatisation was performed to maximise the range of metabolites that can be analysed using GC-MS; i.e., lower boiling point and improve thermal stability. The full procedures for quenching, extraction and derivatisation were undertaken as described previously (Winder et al., 2008) and outlined below.

#### Quenching and extraction

To immediately halt metabolic activities, 15 mL of bacterial culture was quenched with x2 volume of 60% methanol pre-chilled to − 48 °C (30 mL). The quenched samples were immediately centrifuged at 4,347 *g* for 5 min at − 9 °C, after which the supernatant, containing the LB medium and methanol quenching solution, was carefully decanted. The cell pellets were then subjected to a second centrifugation under the same conditions to collect any remaining liquid, which was subsequently removed by pipetting. This procedure minimised residual extracellular medium and quenching solvent prior to storage. The quenched pellets were then kept frozen at − 80 °C, until further analysis. At the point of analysis, upon defrosting the samples, 500 µL of cold (− 48 °C) 80% methanol was added to the quenched biomass to resuspend the pellets and these were transferred to 2 mL microcentrifuge tubes. This step was repeated to minimise the loss of biomass during the transfer. To extract the intracellular metabolites, freeze-thaw cycling approach using liquid nitrogen was performed three times. The cell suspension was then centrifuged at 14,500 *g*, − 9 °C for 10 min prior to biomass normalisation of the sample extracts according to their OD_600nm_. Different volumes of cold (− 48 °C) 80% methanol were added to the biomass-normalised samples to ensure concentration uniformity of all samples. Following this a 700 µL sample volume was taken from each tube. In addition, an equal volume of these normalised extracted samples (250 µL) was collected from each tube, and pooled together to be used as a biological quality control (QC), as detailed in (Broadhurst et al., 2018) and then divided into aliquots of 450 µL. An aliquot (100 µL) of an internal standard (IS, Table S1) was added to the samples, followed by drying using a speed-vac centrifuge (ScanVac MaxiVac Alpha & MaxiVac Beta Vacuum Concentrator, LaboGene, Denmark) for 6 h.

#### Derivatisation

Dried extracts were derivatised with 50 µL of 20 mg/mL O-methoxyamine • HCl dissolved in pyridine, and the samples were vortexed for 10 s prior to heating at 65 °C for 40 min. This was followed by the addition of 50 µL of *N*-Methyl-*N*-(trimethylsilyl)trifluoroacetamide (MSTFA) (Merck Life Science UK Limited, UK) to all the samples, which were then vortexed for 10 s before heating again as described above. Finally, 20 µL of retention index solution (RI, Table S1) was added to each sample, followed by vortexing and centrifugation at 17,000 *g* for 15 min at 4 °C (Muhamadali et al., 2015b; Dunn et al., 2011b; Begley et al. 2009). Aliquots (100 µL) of the derivatised samples were then transferred into GC vials.

### Instrumental setup

A J&W DB-5ms GC Column (Agilent Technologies, Inc.) was used in a split mode (ratio of 20:1) with helium as carrier gas in constant flow rate of 1 mL/min. An Agilent 8890 GC instrument equipped with an Agilent 7250 GC/Q-TOF was operated with an initial temperature program of 70 °C for 4 min. The oven temperature was then increased at 20 °C/min to 300 °C and held for another 4 min before cooling down to 70 °C. The transfer line temperature was 280 °C and a 25 mL/min gas saver flow was switched on after 3 min. The mass range used was 45–600 Da with an acquisition rate of 10 spectra per second (Begley et al., 2009).

### Data analysis

Initially, the GC-MS raw data files were converted into mzML format using ProteoWizard software (64-bit version). The transformed data files were subsequently processed for deconvolution and blank subtraction using Mass Spectrometry-Data Independent AnaLysis software (MS-DIAL version 4.9.2). Deconvoluted data were corrected to remove technical variation using succinic acid *d*_4_, an internal standard, which showed the most reproducibility in terms of standard deviation when compared among the four deuterated standards (mixture of succinic acid *d*_4_, glycine *d*_5_, malonic acid *d*_4_ and alanine *d*_7_, Table S1). Signal drift was further corrected using QC-based LOWESS normalisation. For data curation, first, the relative standard deviation (RSD) of each peak area was calculated from normalised pooled QCs and only those metabolite features with RSD ≤ 30% were kept for further analysis. Second, extracted ion chromatograms (EIC) of each peak were investigated manually to remove those with poor alignment or low signal to noise ratio. Furthermore, the features with a Gaussian chromatographic peak shape were separated and further analysed. These filtering steps were based solely on analytical quality and were independent of statistical hypothesis testing. After preprocessing, high-quality metabolic features were obtained for downstream statistical analysis. Among these curated features, some were annotated as known compounds based on the GC-MS DB-Public-KovatsRI-VS3.msp spectral library, while others were labelled as unknown. Those features that were annotated based on their matching with the database, including, EI spectra and retention index, were considered to be identified to MSI level 2 based on these two orthogonal properties (Sumner et al., 2007).

MetaboAnalyst, an online platform for metabolomics data analysis, was used to analyse pre-processed data by using *t*-test (*p* < 0.05) and ANOVA post-hoc analysis. All chemometric analysis including principal component analysis (PCA) and discriminant function analysis (PC-DFA), were applied using MATLAB^®^ software (version r2019b, The MathWorks Inc., UK); our code is available via GitHub (https://github.com/Biospec/). Significant features identified using *t*-test and ANOVA, together with important annotated metabolites selected from PCA and PC-DFA loadings plots were subsequently combined and investigated. Metabolites from the multivariate analyses were selected based on visual examination of the loadings plots. Specifically, variables exhibiting the largest positive and negative loadings values, which were clearly separated from the main distribution of variables and therefore contributed most strongly to sample discrimination (approximately > + 0.06 and < − 0.07 in the representative PC-DFA loading plot of the CYT *b*_*5*_-producing dataset), were selected for metabolite identification and biological interpretation. The relationship among those annotated metabolites in biological pathways of *E. coli* was determined using pathway analysis from the MetaboAnalyst platform as well as individual evaluation using classical biochemistry pathways and the Kyoto Encyclopedia of Genes and Genomes (KEGG). The pathway analysis parameters were set as follows: the visualisation method was a scatter plot based on testing significant features; the enrichment method applied was the Hypergeometric Test; the topology measure used was Relative-betweenness Centrality; and the reference metabolome included all compounds from the selected pathway library-Prokaryotes, *Escherichia coli* K-12 MG1655 (KEGG).

## Results

### GC-MS data analysis

**Fig. 1:**
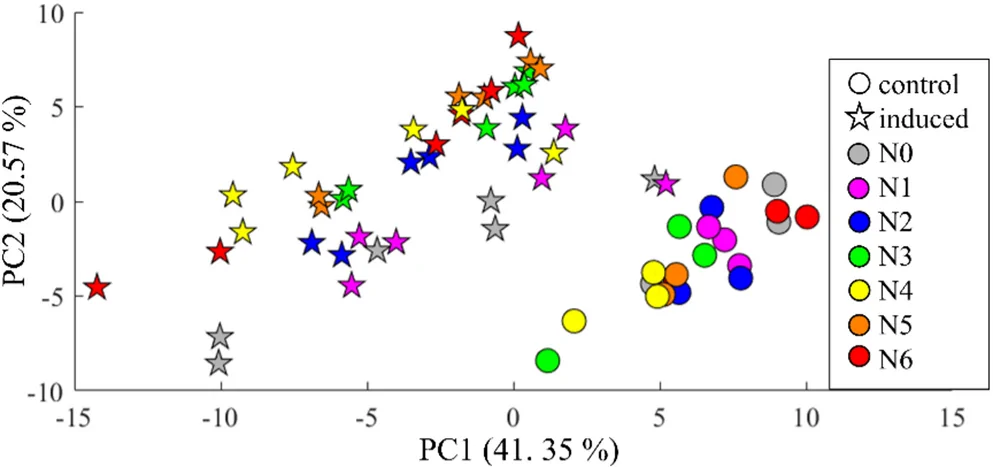
PCA scores plot of the GC-MS metabolic profiles of CYT *b*_*5*_-producing (stars, *n* = 6 biological replicates) and non-producing (circles, *n* = 3 biological replicates) *E. coli* N4830-1 strains from GC-MS metabolic profiles. N0–N6 indicate strains containing 0 to 6 copies of the cyt *b*_*5*_ gene. The number in brackets describes the percentage of the total explained variance (TEV) associated with each PC

In this study, seven recombinant *E. coli* N4830-1 strains (N0–N6) harbouring the pEX-CYT plasmid were investigated. The N4830-1 host strain contains a temperature-inducible λ *c*I857 repressor system, in which a temperature shift to 38.5 °C relieves repression and induces expression of the recombinant CYT *b*_5_ gene. The strains differ in CYT *b*_*5*_ gene copy number, ranging from zero to six copies, and were designated N0 to N6 accordingly. N0 represents the control strain carrying the plasmid backbone without additional CYT *b*_5_ gene copies. The intracellular metabolic profiles of the CYT *b*_5_-producing *E. coli* N4830-1 were investigated using GC-MS. After deconvolution, 861 metabolic features were detected and normalised using the internal standard, succinic acid *d*_4_. These metabolic features were subsequently pre-processed by blank filtration, resulting in 580 features prior to data curation by removal of irreproducible features in the QCs (RSD > 30%). Data were further curated by elimination of those features with non-Gaussian chromatographic peak shape, using the EIC chromatogram, resulting in a total of 340 features being retained. These metabolic features were subsequently subjected to PCA to study and to compare the metabolic changes among the seven bacterial strains in both conditions: under recombinant protein induction at 38.5 °C and control groups at 30 °C. A PCA scores plot of the GC-MS data collected for all the samples was constructed, which illustrated a separation between the control (circles) and induced (stars) samples (Fig. 1). This segregation between the two culture conditions was predominantly according to PC1 axis, which accounted for 41.35% of the total explained variance (TEV).

The first 20 PCs, with a TEV of 92.07%, were next analysed using DFA with *a priori* groups of 14 classes, i.e., seven individual strains and two conditions – as no information on either gene copy or culture conditions was provided to the DFA algorithm, these methods can be considered semi-supervised. This combined process called PC-DFA resulted in a scores plot (Figure S1) of the GC-MS data, which displayed a trajectory similar to that seen in the PCA scores plot (Fig. 1), showing the two clusters of CYT *b*_5_-producing and non-producing *E. coli* strains. Whereas DF1 axis illustrates the distinct cultivation conditions, DF2 axis provided further informative trend showing the separation of strains N0 to N6 from negative to positive (Figure S1). Notably, the results from PC-DFA do not require validation, as the observed trend followed an ordered sequence from strain N0 to N6 without explicitly providing this order to the algorithm. The observed trend is consistent with the increasing number of cyt *b*_*5*_ gene copies (0–6), which is expected to correlate with target protein expression levels; however, contributions from plasmid-associated metabolic burden cannot be excluded as a potential factor influencing the metabolic profiles. However, this response was not strictly linear, indicating that metabolic changes are influenced by regulatory constraints and adaptive stress responses rather than gene copy number alone. Importantly, the metabolic profiles of the uninduced strains and the induced N0 strain did not overlap completely, suggesting that temperature induction itself represents a dominant driver of metabolic reprogramming, while gene copy number further modulates specific metabolic features under induced conditions.

**Fig. 2:**
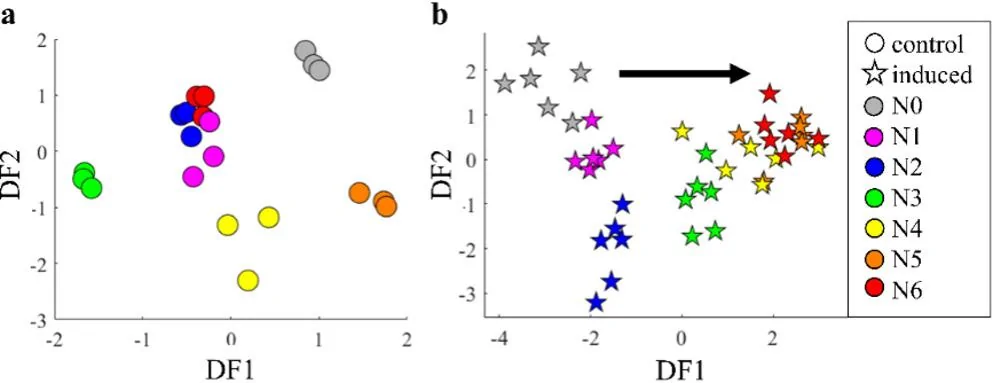
PC-DFA scores plots of *E. coli* N4830-1 strains in **a** control and non-induced conditions (circles, *n* = 3 biological replicates) with 96.53% TEV using PCs 1–15 with the *a prior* class membership based on the biological replicates, and **b** CYT *b*_5_ -producing condition (stars, *n* = 6 biological replicates) with 90.60% TEV using PCs 1–15 with the *a prior* class membership based on the biological replicates. The black arrow shows the trend of induced samples from strains N0-N6

To investigate the metabolic changes within each condition, the data were analysed separately, based on the two culturing conditions (induced cultures or uninduced controls). The PCA scores plot of the induced samples illustrated a trend based on the PC2 axis (14.87% TEV), showing strains N0 and N1 in the negative side, strain N2 on the middle and strain N3-N6 on the positive side (Figure S2b). However, as expected, no clear trends were detected in the PCA scores plot of the control samples (Figure S2a). Furthermore, while the PC-DFA scores plot of the data from the control condition demonstrated no specific trend (Fig. 2a), that of the induced group illustrated separations according to DF1 axis from strain N0 to N6 as shown in Fig. 2b. These trends on both PCA and PC-DFA scores plots of induced samples were further evaluated individually and investigated via pathway analysis in MetaboAnalyst. The input for this analysis were the high-contributing metabolites identified by all chemometrics and statistical tests, as described in the methods section. These are also listed in Tables S2 and S3. MetaboAnalyst enables comprehensive metabolic pathway analysis by integrating pathway enrichment analysis with pathway topology analysis. The results of this analysis suggested that differential levels of CYT *b*_*5*_ production modulated multiple metabolic pathways, including the TCA cycle, glycolysis/gluconeogenesis, pentose phosphate pathway, glyoxylate and dicarboxylate metabolism, purine metabolism, pyrimidine metabolism, and glycerolipid metabolism.

CYT *b*_*5*_ production is regulated by a *λP*_L_ promoter that requires an increase in cultivation temperature (30 to 38.5 °C) to induce gene expression. Therefore, it was necessary to exclude any metabolic effects attributable solely to this 8.5 °C temperature shift, to enable accurate investigation and detection of metabolic changes specifically associated with CYT *b*_*5*_ production. To assess the impact of temperature induction, the bacterial strain N0 (host containing the plasmid but lacking the cyt *b*_*5*_ gene) was analysed under the two culture conditions using PCA (Figure S3) and in combination with *t*-test statistical evaluation. Importantly, the metabolic profiles of the uninduced and induced N0 strain did not fully overlap (Figure S3), indicating that temperature induction is the dominant driver of metabolic reprogramming, with gene copy number contributing additional modulation under induced conditions. The important annotated metabolites listed in Table S4 were thus identified as being influenced exclusively by temperature variation rather than by CYT *b*_*5*_ production.

## Discussion

### GC-MS data interpretation

#### Effect on energy metabolism

**Fig. 3:**
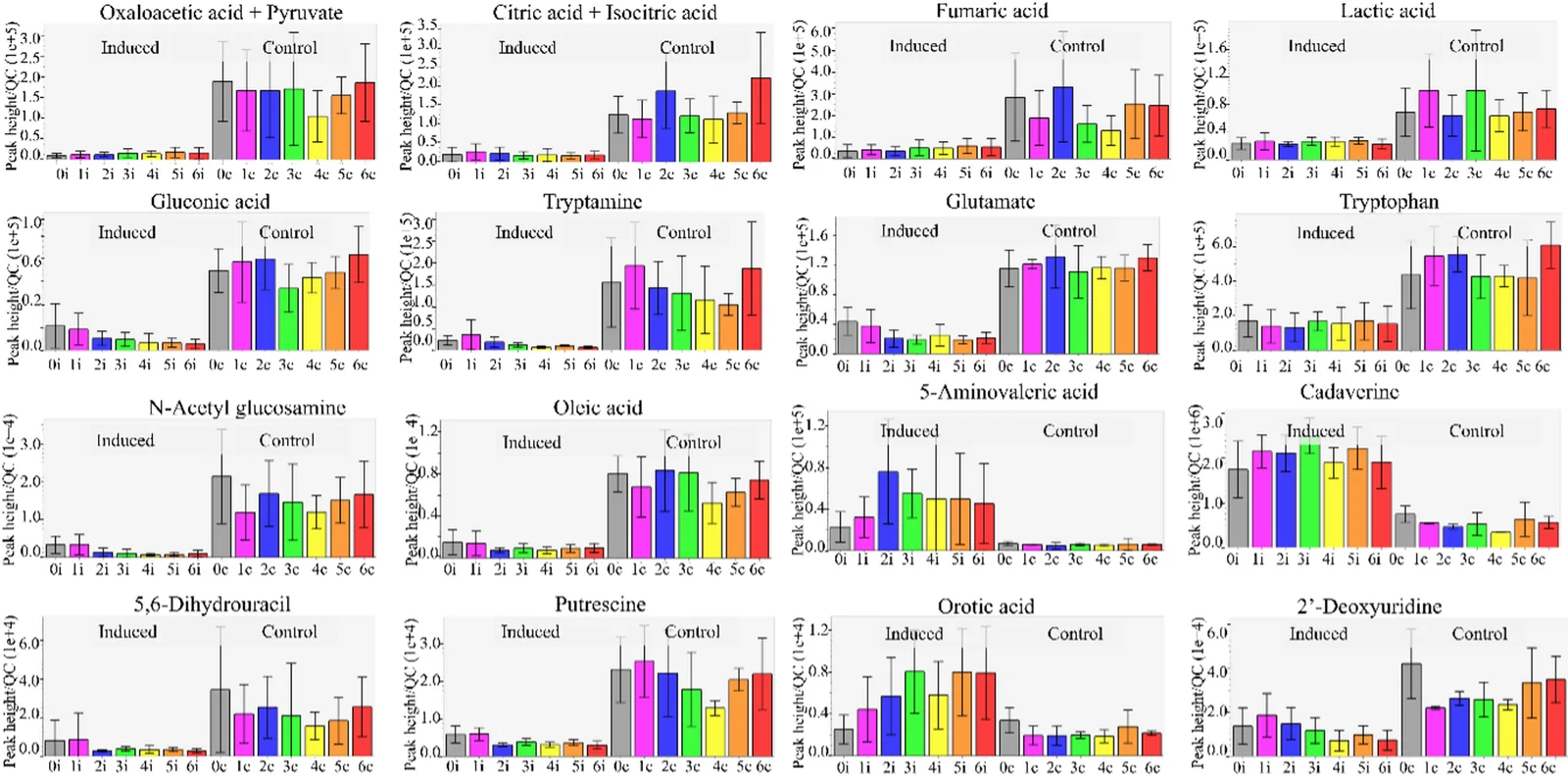
Significant annotated metabolites identified by *t*-test analysis (comparing 2 classes: induced N0-N6 strains (*n* = 6 biological replicates) labelled as 1–7 and control N0-N6 strains (*n* = 3 biological replicates) labelled as 8–14) and chemometrics analyses (PCA and PC-DFA with 14 classes). The bar represents the means and standard deviations are represented by error bars

Regarding the chemometric and statistical analyses, high-contributing metabolic features were mapped to various fundamental metabolic pathways in the bacterial host. This includes central metabolic processes such as glycolysis and TCA cycle. These pathways, in conjunction with the electron transport chain, play a crucial role in cellular energy production. Many metabolic features within these pathways were observed in significantly lower levels in CYT *b*_5_-producing strains compared to the control. This includes metabolites such as pyruvate, oxaloacetate, fumarate, lactate, citrate, isocitrate, gluconic acid, glucuronic acid and 3-phosphoglycerate (Fig. 3).

**Fig. 4:**
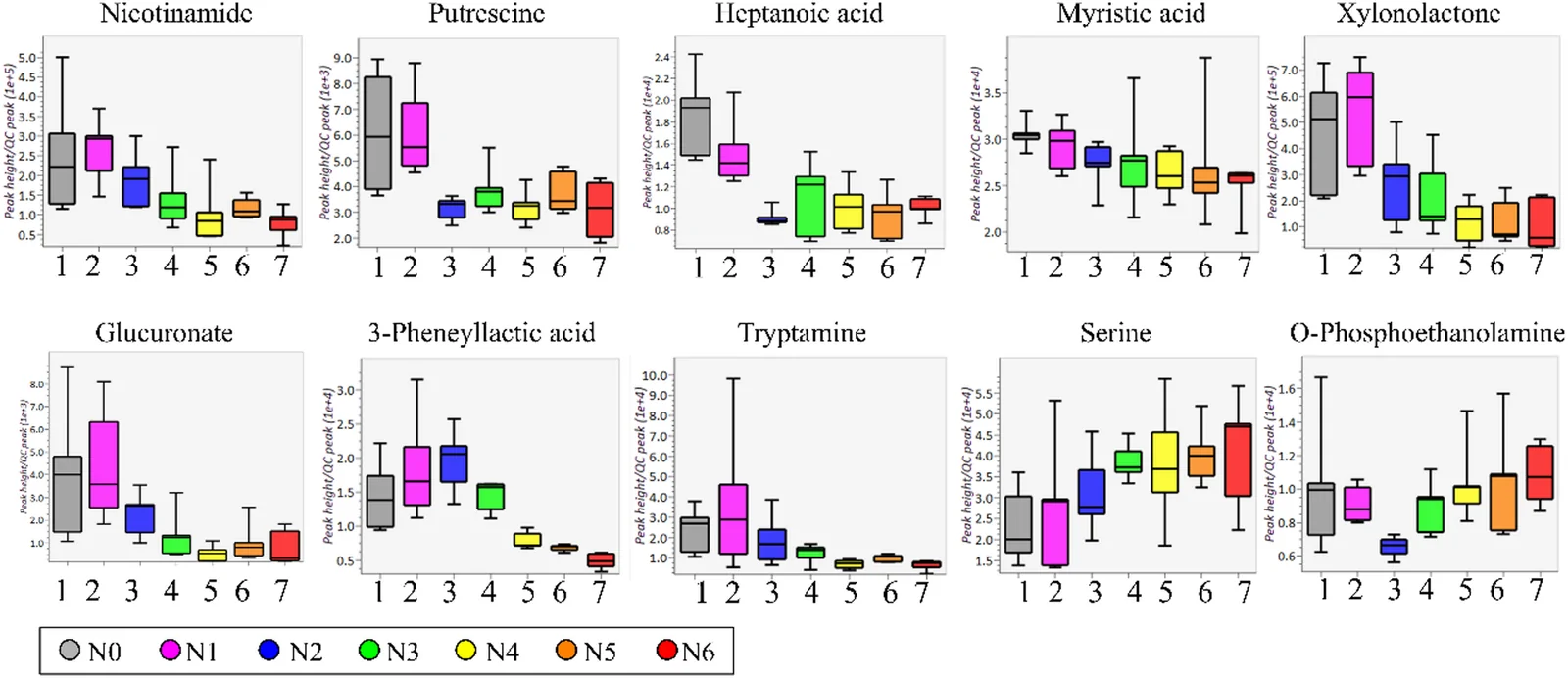
High-contributing metabolites identified by ANOVA (7 classes of 7 strains, *n* = 6 biological replicates) and chemometrics analyses (PCA and PC-DFA with 7 classes, *n* = 6 biological replicates). These plots show box-whiskers where the line within the box is the median value, the top and bottom of the boxes are the 25th and 75th percentiles, the size of the box is the interquartile range (IQR), and the whiskers extend to the most extreme data points

The reduced abundance of these metabolites indicates their substantial utilisation during temperature-induced cultivation, likely reflecting the high energetic demands associated with recombinant protein production. Notably, under induction conditions, nicotinamide was identified as a key metabolite whose concentration progressively declined with increasing copy numbers of the cyt *b*_*5*_ gene across bacterial strains N0 to N4 (Fig. 4). Nicotinamide is a precursor of the essential energy-related cofactor nicotinamide adenine dinucleotide (NAD), and it can be biosynthesised from tryptophan, which was likewise identified as a significant metabolite exhibiting differential levels between the two bacterial culture conditions (Fig. 4).

#### Effect on bacterial stress responses

Several metabolites associated with the pentose phosphate pathway, the glyoxylate shunt, and glutathione metabolism exhibited significant changes in abundance between the induced and control samples. Specifically, there was a decrease in the levels of pyruvate, oxaloacetate, citric acid, isocitric acid, gluconic acid, glutamate, putrescine, and ribose-5-phosphate in the CYT *b*_5_-producing condition. Under this condition, induced samples exhibited a pronounced increase in the levels of cadaverine and 5-aminovaleric acid (5-AVA), metabolites that are likely derived from lysine catabolism. Additional metabolites, including the polyamines cadaverine, putrescine, and spermidine, were also detected. These compounds are known to be upregulated in response to a range of physicochemical stresses, such as elevated temperature, osmotic fluctuations, reactive oxygen species, and ultraviolet radiation (Rhee et al., 2007). Specifically, putrescine concentrations declined under induction, whereas cadaverine displayed an increasing trend.

#### Effect on permeability of bacterial host cells

Numerous intermediates involved in bacterial cell wall and membrane biosynthesis exhibited significant alterations between the two culture conditions. In particular, glycerol, glycerol-3-phosphate, ethanolamine, *O*-phosphoethanolamine, and several unsaturated fatty acids, including oleic acid, elaidic acid, and palmitoleic acid were among the metabolites affected by the temperature-induced production of CYT *b*_5_, serving as intermediates in phospholipid biosynthesis (Sinensky, 1971). Moreover, bacterial cell wall synthesis appeared to be impacted, as evidenced by the significantly reduced levels of *N*-acetylglucosamine in the induced condition compared to the control set.

#### Effect on nucleotide metabolisms

Nucleotide metabolism, specifically purine and pyrimidine metabolism, also displayed alterations under the investigated conditions. While certain metabolites detected in these pathways exhibited a decreasing trend in the CYT *b*_5_-producing condition, such as 5,6-dihydrouracil, 2-deoxyuridine, glutamine, hypoxanthine, and thymine, others such as orotic acid and 3-aminoisobutyric acid showed a significant increase. However, it is worth noting that cytosine and orotic acid from pyrimidine metabolism, and guanine from purine metabolism, exhibited significant alterations under both cultivation conditions (control and induced samples) and strain effects (inducing strains N0-N6). These findings indicate that the metabolic changes likely reflect the combined effects of heat induction and CYT *b*_5_ production.

### Biochemical discussions

Metabolic profiling of the CYT *b*_5_-producing *E. coli* N4830-1 was investigated using GC-MS as this method focusses on central carbon and nitrogen metabolism. The samples were cultured under optimal conditions, first at 30.0 °C, then using an induction temperature of 38.5 °C (for the *λP*_L_ promoter) and an incubation time of 9 h, as determined in our previous publication (Tengsuttiwat et al., 2022). The generated GC-MS data were processed using QC practices (Broadhurst et al., 2018) and then subjected to statistical tests, including *t*-tests and ANOVA, as well as chemometric analyses such as PCA and PC-DFA. The clustering patterns obtained from both PCA and PC-DFA were consistent with those previously reported using FT-IR spectroscopy (Tengsuttiwat et al., 2022). Notably, the PC-DFA scores plots demonstrated a clear gene copy number-dependent trend among induced samples (N0-N6) across three independent batch cultures, underscoring the reproducibility and robustness of the overall analytical workflow. To further distinguish the effect of temperature induction from recombinant protein production, strain N0 (host containing the plasmid backbone but lacking the CYT *b*_5_ gene) was analysed under both culture conditions (30.0 °C and 38.5 °C) using PCA (Figure S3) together with *t*-test-based statistical evaluation. This analysis identified a subset of annotated metabolites (Table S4) that were influenced exclusively by temperature variation rather than CYT *b*_5_ expression. These results demonstrate that the experimental design allows partial separation of temperature-induced metabolic responses from those associated with gene copy number-dependent recombinant protein production. Importantly, the present GC-MS analysis further provided detailed biochemical insights into specific metabolites that play crucial roles under different cultivation conditions and among the seven bacterial strains. The important annotated metabolites were mapped onto metabolic pathways, revealing major effects on energy-related mechanisms, bacterial cell envelope biosynthesis, and nucleotide metabolisms.

The synthesis of recombinant proteins is an energy-intensive process, especially in these strains where CYT *b*_*5*_ production reached 0.73 mM calculated from data in our previous publication (Tengsuttiwat et al., 2022) using the extinction coefficient value of 171 cm^− 1^ mM^− 1^ at 423 nm (Gómez-Tabales et al., 2020). Estimated CYT *b*_*5*_ concentrations for each strain are provided in the Supplementary Information (Table S6). Consistent with this, previous studies have reported a significant decrease in intracellular ATP levels following temperature-induced recombinant production of human fibroblast growth factor protein (Templeton et al., 2013). Additionally, cells producing recombinant proteins also exhibit increased metabolic flux through the TCA cycle and related pathways to meet the elevated ATP demand required for intensive protein synthesis and stress response mechanisms (Wittmann et al., 2007). Consequently, the substantial ATP requirement associated with CYT *b*_*5*_ production likely contributed to the observed depletion of metabolites involved in key energy-generating pathways, including the TCA cycle, glycolysis/gluconeogenesis, the pentose phosphate pathway, as well as glyoxylate and dicarboxylate metabolism. This observation is also supported by prior studies indicating that glycolytic flux in *E. coli* is highly responsive to variations in ATP demand (Koebmann et al., 2002).

During the recombinant protein production, the bacterial host exhibited significant physiological alterations in response to the temperature change between the control and inducing culture conditions. These adaptations prominently involved modifications in cell wall and membrane metabolisms, particularly influencing membrane fluidity, as previously documented in the literature (Russell, 2003; Wang et al., 2023). Membrane integrity in *E. coli* is influenced not only by heat stress but also by environmental factors, including temperature and pH fluctuations (Rowlett et al., 2017). In the present study, an increase in pH from 6.8 to 8.2 was observed following induction (Table S5). Such alkalinisation may be attributed to the oxidation of carbon sources, depletion of amino acids, and the accumulation of basic metabolic by-products (Sánchez-Clemente et al., 2020). Additionally, recombinant protein production imposes a considerable metabolic burden on the host, triggering adaptive responses including rearrangement of lipid composition (Ami et al., 2009), an increase in saturated fatty acids involved in phospholipid metabolism (Sinensky, 1971), and elevated fluxes of pyruvate, acetate, and lactate formation (Wittmann et al., 2007). Collectively, these adaptations reflect coordinated cellular strategies to maintain membrane stability and metabolic balance under the combined stresses of temperature induction and recombinant protein synthesis.

Furthermore, stress-related proteins are often synthesised during recombinant protein production, leading to an increased ATP demand (Hoffmann & Rinas, 2004). Heat shock proteins, for example, can be triggered to cope with the stressful environment (Schumann, 2016). Both the membrane adaptation mentioned above, and the synthesis of stress-responsive proteins are energy-intensive processes. This could lead to significant depletion of glucose for energy production, thereby promoting the utilisation of alternative carbon sources such as acetate commonly associated with recombinant protein synthesis (Han and Eiteman, 2019). This utilisation was evidenced by the observed impact on the glyoxylate shunt due to CYT *b*_5_ production. However, glucose levels remained relatively stable among all the samples. This may be explained by the fact that glucose can also be synthesised from fatty acids through gluconeogenesis and glyoxylate cycle (Cordero et al., 2008). In this process, the glyoxylate cycle provides succinate, which enters the TCA cycle and subsequently feeds into gluconeogenesis, thereby maintaining the detected glucose level. This observation also supports the finding of reduction in unsaturated fatty acids, such as oleic acid, elaidic acid, and palmitoleic acid, in the induced samples. The glyoxylate shunt, along with the pentose phosphate pathway, plays a crucial role in carbon homeostasis. The significant alterations observed in gluconic acid and pyruvate within the pentose phosphate pathway suggest metabolic adaptation to balance carbon flux. When preferred carbon sources like glucose become insufficient to meet increased energetic requirements, *E. coli* can activate the glyoxylate shunt to utilise simpler carbon sources such as acetate and fatty acids.

Additionally, oxidative stress is a common consequence of recombinant protein production due to increased cellular energy demand (Templeton et al., 2013). In response, bacterial cells may utilise pathways such as the pentose phosphate pathway, glyoxylate shunt, and glutathione metabolism to mitigate the effects of such stress (Stincone et al., 2015; Ahn et al., 2016; Ozkul & Kocak, 2020). The pentose phosphate pathway is essential for supplying precursors for nucleotide and amino acid biosynthesis. Correspondingly, significant alterations in metabolites such as cytosine, orotic acid, *O*-phosphoethanolamine, myristic acid, heptanoic acid, serine, and tryptamine across the seven CYT *b*_5_-producing strains suggest that these biosynthetic pathways were affected by heat-induced CYT *b*_5_ expression and potential differences in cyt *b*_5_ gene copy number (Fig. 4).

Furthermore, the untargeted GC-MS metabolomics approach used in this study does not allow absolute quantification of metabolites (rather a peak ratio against an internal standard). Although the quenching protocol incorporated two consecutive centrifugation steps to minimise extracellular medium carryover, the absence of a dedicated washing step means that complete removal of extracellular metabolites cannot be guaranteed. Consequently, some detected compounds, such as amino acids, may originate from either endogenous synthesis or uptake from the nutrient-rich LB medium. Future studies incorporating metabolic footprinting would help clarify metabolite origins and consumption dynamics.

## Conclusions

In this study, an untargeted metabolomic approach was employed utilising GC-MS to examine the metabolic profiles of seven strains of *E. coli* N4830-1, in the context of temperature-induced production of CYT *b*_5_. The most notable alterations were observed in the TCA cycle, where key metabolites such as pyruvate, oxaloacetate, citrate, isocitrate, and fumarate exhibited marked depletion in the induced condition. This observation indicates the heightened energy requirements inherent to recombinant protein production systems. The imposition of heat stress and the demands of recombinant protein production necessitate metabolic adaptations within bacterial hosts. Significant alterations in detected metabolites suggest responses aimed at fortifying bacterial cell wall and membrane biosynthesis, as well as deploying defensive mechanisms against oxidative stress through pathways such as the pentose phosphate pathway, glyoxylate shunt, and glutathione metabolism. The aforementioned mechanisms all entail energy expenditure, thereby increasing the demand for energy within host cells. However, to gain a comprehensive understanding of the metabolic burden associated with recombinant CYT *b*_5_ production among the seven strains, future studies could explore a combination of metabolic profiling (intracellular metabolites), footprinting (extracellular metabolites), and lipidomics (membrane lipids). Additionally, targeted metabolomics approach could be employed to facilitate quantitative analysis. Since energy consumption played a crucial role among all the samples, adenylate energy charge is worth considering as it represents the available metabolic energy of a host at the time of sampling (Atkinson & Walton, 1967; Atkinson, 1968). Furthermore, fluxomics is a valuable metabolomic approach for monitoring metabolic pathways (Emwas et al., 2022); for instance, the analysis of metabolic flux using ^13^C-labeled molecules. Such investigations may reveal potential bottlenecks in the recombinant protein production process and support optimisation of the production system, but only really work in highly defined medium with a sole carbon supply which may affect recombinant protein production as current production systems are replete with nutrients. Subsequent genetic engineering strategies or media optimisation, for example, could be proposed to enhance the efficiency of the production system.

In conclusion, this study shows that CYT *b*_5_ production triggers coordinated metabolic changes that impact energy metabolism, cell wall and membrane biosynthesis, nucleotide metabolism, and bacterial stress responses. These insights may in the future offer valuable guidance for optimising recombinant protein production processes in prokaryotic hosts.

## Supplementary Information

Below is the link to the electronic supplementary material.

Supplementary Material 1

## Data Availability

GC-MS data are freely available at MetaboLights (Yurekten et al. 2023) repository with the unique identifier MTBLS15018 (www.ebi.ac.uk/metabolights/).
