# Supplementary material for: Metabolic profiling analysis of cytochrome b5 production in E. coli N4830-1 using GC-MS
Source: Metabolomics. 2026 Sep 22;22(5):155. doi: 10.1007/s11306-026-02522-5 (PMC13597647; doi:10.1007/s11306-026-02522-5)
Supplement: Supplementary file 1 [file 11306_2026_2522_MOESM1_ESM.docx]

**supplementary information**

Table S1. Media and solutions used in this study with their ingredients.

| **Media/Solutions** | **Ingredients** |
| --- | --- |
| Internal standard (IS) | 0.017 mg/mL succinic acid *d*_4_  0.017 mg/mL glycine *d*_5_  0.034 mg/mL malonic acid *d*_4_  0.17 mg/mL alanine *d*_7_  In H_2_O |
| Retention index (RI)  RI stock solution  RI working solution | 40 µL decane  40 µL dodecane  40 µL pentadecane  30 mg nonadecane  30 mg docosane  In 10 mL hexane  1 mL RI stock solution  9 mL anhydrous pyridine |

Figure S1. PC-DFA scores plot of CYT *b*_5_-producing (stars, *n* = 6 biological replicates) and non-producing (circles, *n* = 3 biological replicates) *E. coli* N4830-1 strains from GC-MS metabolic profile. The number of PC used to generate this was1-20 and these PCs accounted for 92.07 % of the total explain variance. The *a priori* class structure was based on the biological replicates.

Figure S2. PCA scores plots of *E. coli* N4830-1 strains in (a) control or non-induced condition (circles, *n* = 3 biological replicates) and (b) CYT *b*_5_-producing condition (stars, *n* = 6 biological replicates). The total explained variance of each PC is provided within the axes labels.

Figure S3. PCA scores plots of *E. coli* N4830-1 strain N0 in control (circles, *n* = 3 biological replicates) and induced (stars, *n* = 6 biological replicates) conditions. The total explained variance of each PC is provided within the axes labels.

Table S2. Lists of important annotated metabolites of all bacterial samples from *t*-test (control *versus* induced samples), PCA and PC-DFA (20 PCs, 92.07 % TEV).

| **Accurate mass** | **Retention index** | **Identifier** | **Significant test** |
| --- | --- | --- | --- |
| 204.1017 | 1825.17 | (-)-Shikimic acid | PC-DFA |
| 260.1521 | 1735.46 | 2-Aminoadipic acid | PCA, *t*-test, PC-DFA |
| 174.1145 | 1457.33 | 3-Aminoisobutyric acid | PC-DFA |
| 193.1063 | 1609.21 | 3-Phenyllactic acid | PC-DFA |
| 243.1001 | 1505.58 | 5,6-Dihydrouracil | PCA |
| 174.1144 | 1636.68 | 5-Aminovaleric acid | PCA, *t*-test, PC-DFA |
| 174.1145 | 2432.02 | 5-Methoxytryptamine | PCA |
| 116.0902 | 1118.93 | Alanine | PC-DFA |
| 116.0902 | 1624.63 | Alanylalanine | PC-DFA |
| 174.1146 | 1859.96 | Cadaverine | PCA, *t*-test, PC-DFA |
| 273.1002 | 1842.54 | Citric acid+Isocitric | PCA, *t*-test, PC-DFA |
| 254.1163 | 1541.82 | Cytosine | PC-DFA |
| 361.1718 | 2744.17 | D-(+)-Maltose | PCA |
| 117.0378 | 2217.74 | Elaidic acid | PCA, *t*-test, PC-DFA |
| 174.1145 | 1247.98 | Ethanolamine | PCA |
| 245.0684 | 1359.81 | Fumaric acid | PCA, PC-DFA |
| 159.0965 | 1977.29 | Galactosamine | PC-DFA |
| 333.1403 | 2045.47 | Gluconic acid | PCA, *t*-test, PC-DFA |
| 333.1403 | 1976.38 | Glucuronate | PCA, *t*-test, PC-DFA |

Table S2. Lists of important annotated metabolites of all bacterial samples from *t*-test (control *versus* induced samples), PCA and PC-DFA (20 PCs, 92.07 % TEV) (cont.).

| **Accurate mass** | **Retention index** | **Identifier** | **Significant test** |
| --- | --- | --- | --- |
| 84.04523 | 1539.71 | Glutamate | PCA, *t*-test, PC-DFA |
| 156.0856 | 1794.09 | Glutamine | PC-DFA |
| 205.1093 | 1291.58 | Glycerol | PC-DFA |
| 243.0657 | 1753.14 | Glycerol-3-phosphate + Glycerol-2-phosphate | PC-DFA |
| 174.1151 | 1859.96 | Gly-Gly | PCA, *t*-test, PC-DFA |
| 352.1478 | 2150.62 | Guanine | PC-DFA |
| 187.1168 | 1174.48 | Heptanoic acid | *t*-test, PC-DFA |
| 158.1373 | 1290.7 | Isoleucine | PC-DFA |
| 117.0742 | 1076.34 | Lactic acid | *t*-test |
| 218.1411 | 1463.42 | L-Homoserine | PCA |
| 192.1557 | 1380.81 | L-Serine | PCA, PC-DFA |
| 202.1066 | 2227.19 | L-Tryptophan | PCA, *t*-test |
| 202.1154 | 2120.45 | *N*-Acetyl glucosamine | PCA, *t*-test, PC-DFA |
| 84.0452 | 1800.33 | *N*-Acetyl glutamate | PCA, *t*-test, PC-DFA |
| 179.0656 | 1491.25 | Nicotinamide | PC-DFA |
| 243.1257 | 2216.73 | Oleic acid | PCA, *t*-test, PC-DFA |
| 299.0741 | 1811.54 | *O*-Phosphoethanolamine | PC-DFA |
| 254.0924 | 1766.5 | Orotic acid | PCA |
| 174.0598 | 1063.88 | Oxalacetic acid+Pyruvate | PCA, *t*-test, PC-DFA |
| 117.0378 | 2033.47 | Palmitoleic acid | *t*-test, PC-DFA |
| 299.0741 | 1294.08 | Phosphate | PC-DFA |
| 142.1062 | 1318.9 | Proline | PCA, PC-DFA |

Table S3. Lists of important annotated metabolites of inducing samples from ANOVA (seven bacterial strains), PCA and PC-DFA (15 PCs, 90.60 % TEV).

| **Accurate mass** | **Retention index** | **Identifier** | **Significant test** |
| --- | --- | --- | --- |
| 260.1521 | 1735.46 | 2-Aminoadipic acid | ANOVA |
| 193.1063 | 1609.21 | 3-Phenyllactic acid | PCA, ANOVA, PC-DFA |
| 299.0742 | 1787.53 | 3-Phosphoglycerate | PCA |
| 174.1145 | 2432.02 | 5-Methoxytryptamine | PCA, ANOVA |
| 264.1123 | 1890.32 | Adenine | PC-DFA |
| 116.0902 | 1118.93 | Alanine | PC-DFA |
| 254.1163 | 1541.82 | Cytosine | PC-DFA |
| 117.0378 | 2217.74 | Elaidic acid | ANOVA, PC-DFA |
| 333.1403 | 2045.47 | Gluconic acid | PCA |
| 333.1403 | 1976.38 | Glucuronate | PCA, ANOVA, PC-DFA |
| 243.0657 | 1753.14 | Glycerol 3-phosphate + Glycerol-2-phosphate | PC-DFA |
| 352.1478 | 2150.62 | Guanine | PC-DFA |
| 187.1168 | 1174.48 | Heptanoic acid | PC-DFA |
| 158.1373 | 1290.7 | Isoleucine | PC-DFA |
| 192.1557 | 1380.81 | L-Serine | PCA |
| 179.0656 | 1491.25 | Nicotinamide | PCA, ANOVA |
| 299.0741 | 1811.54 | *O*-Phosphoethanolamine | PC-DFA |
| 254.0924 | 1766.5 | Orotic acid | PCA, ANOVA |
| 117.0378 | 2033.47 | Palmitoleic acid | ANOVA, PC-DFA |
| 299.0741 | 1294.08 | Phosphate | PC-DFA |
| 174.1144 | 1650.81 | Putrescine | ANOVA |
| 230.1778 | 1521.7 | Trans-4-hydroxy-L-proline | PCA |
| 174.1144 | 2398.5 | Tryptamine | PCA, ANOVA, PC-DFA |
| 217.1098 | 1605.95 | Xylonolactone | PCA, ANOVA, PC-DFA |

Table S4. Lists of the top most important annotated metabolites of *E. coli* N4830-1 strain N0 in control and induced conditions from PCA and *t*-test.

| **PCA** | ***t*-test** | **Summary** |
| --- | --- | --- |
| 2-Aminoadipic acid | 2-Aminoadipic acid | 2-Aminoadipic acid |
| 3-Aminoisobutyric acid | Elaidic acid | 3-Aminoisobutyric acid |
| 3-Phenyllactic acid | Heptanoic acid (7:0) | 3-Phenyllactic acid |
| Cadaverine | Oxalacetic acid | Cadaverine |
| Fumaric acid | Palmitic acid (16:0) | Elaidic acid |
| Glutamate | Putrescine | Fumaric acid |
| Gly-Gly | Pyruvate | Glutamate |
| Histidine | Trans-4-hydroxy-L-proline | Gly-Gly |
| L-Serine | Tryptamine | Heptanoic acid (7:0) |
| *O*-Phosphoethanolamine |  | Histidine |
| Oxalacetic acid |  | L-Serine |
| Palmitic acid (16:0) |  | *O*-Phosphoethanolamine |
| Pyruvate |  | Oxalacetic acid |
| Sucrose |  | Palmitic acid (16:0) |
| Tryptamine |  | Putrescine |
|  |  | Pyruvate |
|  |  | Sucrose |
|  |  | Trans-4-hydroxy-L-proline |
|  |  | Tryptamine |

Table S5. The bacterial pH of both before and after induction for all strains.

| **Bacterial strain** | | **N0** | **N1** | **N2** | **N3** | **N4** | **N5** | **N6** |
| --- | --- | --- | --- | --- | --- | --- | --- | --- |
| **pH** | initial | 6.8 | 6.8 | 6.8 | 6.8 | 6.8 | 6.8 | 6.8 |
|  | final | 8.1 | 8.2 | 8.0 | 8.1 | 8.2 | 8.1 | 8.2 |

Table S6. Calculated CYT *b_5_* concentrations for recombinant *E. coli* strains N0-N6 based on the Soret peak absorbance at 423 nm of cell lysates supplemented with 60 µM hemin. Absorbance data were obtained from Tengsuttiwat *et al*. (2022). Data are presented as mean ± SD.

| **Bacterial strain** | **N0** | **N1** | **N2** | **N3** | **N4** | **N5** | **N6** |
| --- | --- | --- | --- | --- | --- | --- | --- |
| Mean absorbance at 423 nm | -0.019 | 1.912 | 3.146 | 3.720 | 4.204 | 4.257 | 4.282 |
| Calculated CYT *b_5_* concentration (mM, mean ± SD) | 0.00 ±0.007 | 0.33 ±0.052 | 0.54 ±0.087 | 0.63 ±0.097 | 0.72 ±0.144 | 0.73 ±0.123 | 0.73 ±0.119 |
